# Do We Need Biomarkers for Disc Degeneration?

**Published:** 2007-02-07

**Authors:** Helen E. Gruber, Edward N. Hanley

**Affiliations:** Department of Orthopaedic Surgery, Carolinas Medical Center, Charlotte, N.C

**Keywords:** disc degeneration, low back pain, biomarkers, discogenic pain

## Abstract

Disc degeneration plays a major role in this country’s medical, social and economic structure. The life-time prevalence of low back pain, which has disc degeneration as its cause, is about 80% in the general population. It is a primary cause of disability and estimated costs related to low back disorders exceed $100 billion per year in the U.S. alone. Biomarkers are becoming increasingly important as indicators of the presence of disease, and in evaluating outcomes during clinical treatment. Cell-based biologic therapies which are currently being developed to treat disc degeneration are going to be most efficacious when applied to the *early stage*s of disc disease. In this article we ask: 1) Whether there are existing biomarkers which could play a role in detecting early stages of disc degeneration, and 2) Highlight exciting potentials in future biomarker screening for disc degeneration.

## Introduction

The NIH Biomarker Definitions Working Group has provided a very useful definition of a biomarker, describing it as “a characteristic that can be measured and evaluated as an indicator of normal biologic processes, pathologic processes or pharmacologic responses to therapeutic intervention” (Biomarkers Definitions Working Group, Bethesda, MD). The Working Group further stated that the use of biomarkers can be quite wide, including evaluation of efficacy and safety (in either *in vitro* studies, *in vivo* animal studies, or early clinical trials), identification of patients with a disease or abnormal condition, characterization of disease stages, indicators of disease prognosis, and in monitoring the clinical response to a disease intervention.

Interest in biomarkers continues to expand today, with application of biomarker detection and measurement capabilities being channeled into practical diagnostic and therapeutic information modalities, and sensitive, specific biomarkers for early disease detection (Liszewski, 2006). Articles in the scientific press reflect a general interest in biomarker applications in early drug development, and protein and pharmacodynamic and metabolomic biomarkers are being developed and marketed. Gene expression techniques have now been utilized in identification of biomarkers, as shown by the recent work by Laterze et al. suggesting discovery of potential new biomarkers for brain injury (Rifa et al. 2006; Laterza et al. 2006). Another novel approach to biomarker identification was recently announced by Gustavson et al. from their work with measurement of the protein concentrations of thymidylate synthase within specific cells and cell compartments in colorectal carcinomas (Gustavson, 2006).

When single biomarkers cannot provide adequate information or specificity, “biomarker profiles” or “biomarker panels” are now commonly being used, and for some diseases we now know that early biomarkers for disease may differ from important biomarkers exhibited during later disease progression.

## High Clinical Relevance of Disc Degeneration

Disc degeneration is a multifactorial process which is influenced by contributions from genetic predisposition, lifestyle conditions (including obesity, occupation, smoking, alcohol consumption), other health factors (such as diabetes), and the aging phenomena. An understanding of the etiology and epidemiology of disc degeneration and low back pain, and of the operative and non-operative options available to the orthopaedic surgeon, are important factors which guide the surgeon in selection of the best treatment for the individual patient (Hanley and David, 1999).

Disc degeneration is a chronic condition associated with morbidity and a significant reduction in the quality of life for the patient. Although not lethal, the socioeconomic consequences of long-term low back pain are high, and, as recently pointed out by Katz, the current estimate for the total costs of low back pain in the United States exceeds $100 billion per year (Katz, 2006). This startlingly high figure points to the critical importance of development of new strategies designed to prevent disc degeneration and low back pain. Many clinical investigators, including our group, feel that cell-based biologic therapies offer great potential for the treatment of disc degeneration (Gruber et al. 2006; Gruber et al. 2001; Gruber et al. 2004). Such therapeutic modalities, which include disc cell augmentation, direction of adult mesenchymal stem cells to a disc-like phenotype, application of growth factors/cytokines, and possibly gene therapy, will be most efficacious when applied to the *early stage*s of disc degeneration. Thus the potential application of biomarkers for early identification of the patient at risk for disc degeneration and low back pain, is an important goal for today’s disc research lab.

## Disc Degeneration and Biomarkers

In late 2005, the NIH, the American Academy of Orthopaedic Surgeons and the Orthopaedic Research Society organized a workshop focused upon disc degeneration. The relevance of biologic markers of matrix formation and matrix turnover in the diagnosis of other musculoskeletal conditions, such as osteoarthritis and osteoporosis, was discussion by Poole (Poole, 2006). Since that presentation, there is now new evidence that serum levels of hyaluronic acid show potential as a biomarker for osteoarthritis based on the findings of Elliott et al. in the Johnston County Osteoarthritis Study (Elliott et al. 2005). However, the avascular nature of the adult disc presents a major block to the employment of the commonly used cartilage and bone biomarkers for studies of the disc. The number of blood vessels in the annulus and the number of vascular canals in the cartilage end plates decrease progressive during childhood (Taylor and Twomey, 1988) and are markedly diminished by age 3 years. By young adulthood, the disc is avascular and cellular nutrition relies on diffusion of solutes through the matrix via disc compression (Maroudas, 1988).

Balaguè et al. have recently carried out a study on the potential value of blood biomarkers in disc metabolism in patients with sciatica (Balagué et al. 2006). Keratan sulfate, hyaluronan and cartilage oligomeric matrix protein (COMP) were tested for their utility as biomarkers. Their study found that a single measurement of these molecules did not have any diagnostic or therapeutic relevance in their subjects with acute radicular compression. All molecules showed an increase after their average 4.3 year follow-up.

In another study, Yüceer et al. investigated the possibility of using changes in serum immunoglobulin concentrations to assess lumbar disc disease (Yüceer et al. 2000). This study of IgG, IgA and IgM measurement found no significant difference in serum levels of patients with disc disease compared to normal controls.

Patients with herniated discs have been found to have increased concentrations of neurofilament protein and S-100 in cerebrospinal fluid (indicating possible damage of axons and Schwann cells in nerve roots) (Brisby et al. 1999); however, it is unlikely that cerebrospinal fluid sampling would be a useful biomarker in the general population.

## Future Directions for Biomarkers for Early Disc Degeneration

In light of its public health importance, we suggest that research must continue in search of relevant, accurate and sensitive biomarkers of disc degeneration. MRI and radiographic imaging techniques certainly provide sensitive and accurate techniques for identification of late-stage disc degeneration based upon the disc morphology images, disc height and hydration. To date, early changes cannot be detected with use of these routine methodologies.

New studies, however, point to exciting future possibilities for application of non-invasive T_1_*_ρ_*-weighted magnetic resonance imaging (MRI) imaging techniques to detect each loss of proteoglycan in the nucleus of the disc (Johannessen et al. 2005). Johannessen et al. found that T_1_*_ρ_* bore a better relationship to sulfated proteoglycans in the disc than did T_2_ imaging results. Other advantages of the T_1_*_ρ_* methodology are that it does not require a contrast agent, it can be performed quickly, and provides a spatial map of proteoglycan content. Majumdar et al. also noted that in addition to the proteoglycan content, the T_1_*_ρ_* findings are also related to water content (Majumdar et al. 2005). In their study, HR-MAS (spectroscopic) data correlated well with the Thompson grade for disc degeneration scoring (Thompson et al. 1990); the researchers also noted that an increase was found for levels of unbound hydroxyprolines and glycine in the annulus which was directly associated with collagen breakdown during disc degeneration.

Biomarkers which could be used to correlate the patient’s status with regard to pain, symptoms and prognosis would be especially valuable. Similarly, imaging studies which might be able to be related to discogenic low back pain would clinically be valuable to assess symptom severity. Although currently of more interest to researchers, biomarkers of pain might be of high value in early stages of disc degeneration when routine imaging studies are asymptomatic.

In conclusion, we hope that we have shown the reader the exciting potentials which development of biomarkers for disc degeneration, especially disc degeneration in its early stages, holds for the future. Important summary points are that:

▪ Disc degeneration poses large heath care and socio-economic costs.
▪ Future cell-based biologic therapies hold great promise for disc degeneration, and may be most useful in cases of early disc degeneration.
▪ Thus identification and development of sensitive and accurate biomarkers for early disc degeneration is an important health care goal.
▪ Serum and urine biomarkers—which work well for osteoarthritis and metabolic bone diseases such as osteoporosis and Paget’s disease – need to be investigated for disc degeneration biomarkers; however such assays may have limited applicability because of the avascular nature of the adult human disc.
▪ Newly developed advances in MRI imaging, which can quantitatively assess tissue hydration in the disc (such as T_1_*_ρ_*) and spectroscopic (HRMAS) methods currently hold exciting promise for useful, non-invasive biomarkers of early disc degeneration.
▪ Development of future biomarkers for discogenic pain, and study of their correlation with severity of symptoms and new imaging results, also holds great promise.
